# Optimization of Fermented Maize Stover for the Fattening Phase of Geese: Effect on Production Performance and Gut Microflora

**DOI:** 10.3390/ani14030433

**Published:** 2024-01-29

**Authors:** Xiaoqing Hong, Yonghong Zhang, Hongyu Ni, Qingxing Xiao, Yijing Yin, Jing Ren, Puze Zhao, Ziyi Zhang, Xiaohui Li, Yumei Li, Yuwei Yang

**Affiliations:** 1 College of Animal Science, Jilin University, Changchun 130062, China; hongxq22@mails.jlu.edu.cn (X.H.); yonghong@jlu.edu.cn (Y.Z.); nihy23@mails.jlu.edu.cn (H.N.); qxxiao22@mails.jlu.edu.cn (Q.X.); yinyj21@mails.jlu.edu.cn (Y.Y.); renj21@mails.jlu.edu.cn (J.R.); pzzhao22@mails.jlu.edu.cn (P.Z.); ziyiz22@mails.jlu.edu.cn (Z.Z.); 2 Center of Animal Experiment, College of Basic Medical Sciences, Jilin University, Changchun 130021, China; lixiaohui@jlu.edu.cn

**Keywords:** geese, fermented maize stover, production performance, intestinal morphology, gut microflora function

## Abstract

**Simple Summary:**

16S rRNA sequencing technology was used to study the diversity and structural composition of gut microorganisms in fattening geese fed with an optimal ratio of FMS. This study reveals the diversity changes in the gut microbiota ecosystem of geese fed with FMS while analyzing the dominant microbial communities and predicting their potential functions. These findings will help to improve the utilization rate of FMS in poultry during the fattening period, reduce feed costs, improve economic benefits and maximize nutritional value.

**Abstract:**

To optimize the utilization of fermented maize stover (FMS) feed during the fattening phase of Xianghai flying geese (XFG), a total of 300 XFG at 125 days of age were randomly assigned to four dietary treatment groups with three replicates of 25 in each set. Group A was fed the basal fattening diet, while the B, C, and D groups were fed the basic fattening diet and diets supplemented with 5%, 10% or 15% FMS, respectively. The findings indicate that the production performance indicators (especially the dressed, eviscerated and breast muscle yield) of Group D closely resembled Group A more than Groups B and C. Intestinal morphometry found that the jejunal villus height and the villus height/crypt depth were significantly increased in Group D compared to Group A. Next, 16S rRNA amplicon sequencing of the extracted DNA revealed that beneficial microbiota (*Coprococcus* and *Victivallis*) showed increased abundance in Group D. Cecal flora function analysis further revealed that some amino acid and glycerol biosynthesis were found to be associated with growth performance in geese. These findings suggest that incorporating 15% FMS as a substitute for a portion of the feed during the fattening phase of XFG can effectively sustain their production performance, optimize the gut microbial community and morphometrical traits, provide new insight into using non-conventional feed resources to reduce feed cost and improve economic benefits in the breeding industry.

## 1. Introduction

China is the largest goose-producing and -consuming country in the world. The goose farming industry incurs significant expenses primarily attributed to feed, with feed costs constituting approximately 70% of stocking expenses [1]. The scarcity of raw feed materials has resulted in persistent price hikes, impeding the goose industry’s growth rate and expansion. To mitigate this pressing issue, exploring alternative feed sources, such as wheat bran, soybean residue and stover [2], which can be incorporated into livestock feed formulations, is imperative. This strategy would alleviate competition between human and animal consumption and diminish the financial burden of animal feeding. The study shows that alfalfa flavonoids are commonly used as a feed additive in broiler diets to improve meat quality and production performance [3]. Similarly, the addition of 5% cassava foliage can enhance feed digestion and improve the meat quality of geese [4]. The output of fermented maize stover (FMS) is the most produced, with an annual production of around one billion tons of maize stover worldwide [5,6]. Research has revealed that through the fermentation process, the crude protein content of corn straw is enhanced, accompanied by augmentation in the presence of advantageous additives such as organic acids, amino acids, phenols and vitamins [7]. Recently, the food industry has studied silage maize for its positive results and certain advantages [8]. Thus, the proper utilization of FMS in animal feed may reduce pollution emissions, address the existing scarcity of feed materials and enhance production and economic outcomes in the field of animal husbandry.

As herbivorous poultry and compared to other avian species, geese have relatively well-developed paired cecums that use fibrous materials through microorganisms [9]. Cellulose-available microorganisms have commendable utilization and adaptability to roughage, such as maize stover [10], enabling the conversion of cellulose into short-chain fatty acids that serve as a source of energy for geese [11]. Studies have shown that the diversity and composition of the gut microbiome in poultry are impacted by diet [12]. For example, diets containing up to 10% lucerne may enhance intestinal barrier function, improve bacteria bonding in Beijing-You chickens and boost the multiplication of beneficial bacteria such as *Lactobacillus* [13]. In addition, a reduced amount of *Bacteroidetes* and an increased amount of *Firmicutes*, which are normally beneficial bacteria dominating the gut, have been observed in geese fed high-fiber diets [14,15]. However, reports on the FMS effects on the gut microbiome and nutrient digestibility in geese are scarce, especially in the fattening period.

Therefore, Xianghai flying geese (XFG) were used as the experimental model in this study. It is a distinctive breed within the breeding sector of Tongyu County that possesses notable traits, including rough feeding resistance, strong disease resistance and characteristics of delicious meat. The purpose of this study was to explore the effects of FMS on production performance, intestinal morphology and the cecal microflora of XFG by replacing different proportions of FMS in basic diets and conducting short-term concentrated fattening for XFG during the fattening period. Alternatively, shortening the fattening period of XFG and using non-traditional feedstuffs to replace part of the feed can reduce feed farming costs, which provides a certain theoretical basis and practical reference value for agricultural and rural animal farmers.

## 2. Materials and Methods

### 2.1. Animals, Diet and Experimental Design

All Xianghai flying geese (XFG) were provided by Jilin Province Jiuzhou Fei Goose Animal Husbandry Technology Co., Ltd. (Baicheng, China). Animal ethics were approved by the College of Animal Science of Jilin University Ethics Committee (SY202306052).

A total of 300 125-day-old healthy XFG with similar body weights were randomly divided into four groups consisting of three replicates with 25 birds per replicate. Group A was fed a basal fattening diet (the control), while the B, C, and D groups were fed a basal fattening diet supplemented with 5%, 10% or 15% fermented maize stover, respectively. The geese house was decontaminated and disinfected before the geese moved in to ensure minimal bacterial contamination. The geese were bred using ground feeding and allowed free movement and exposure to natural sunlight and air. The formulations and nutritional levels of the basal fattening diet and fermented maize stover are formulated in Table 1. The experiment duration was 28 days in total; the first 7 days were a pre-test period for the geese to adapt to the basal fattening diet, and the formal trial period lasted for 21 days.

### 2.2. Preparation of Fermented Maize Stover and Determination of Fermentation Quality

Fresh maize stover (60% moisture content) harvested from the agricultural trial site of Jilin University was cut into 1–2 cm lengths and packed in plastic bags (five bags in total, each bag containing 30 kg) for sealing. After 45 d of fermentation at room temperature, the bags were opened and used to determine fermentation quality. We obtained 4 g of silage samples (20 g in total) from each bag and added 180 mL of distilled water. The homogenate was filtered through four layers of gauze 24 h later to prepare the silage leachate. A precision pH meter (PHS-3C) analyzed the filtrate. Lactic acid, acetic acid, propionic acid and butyric acid contents were analyzed using high-performance liquid chromatography (HPLC) with SHIMADZE-10A (Shimadze, Kyoto, Japan). The concentration of NH_3_-N was determined using a colorimetric method of phenol–sodium hypochlorite.

### 2.3. Sample Collection

At the end of the experimental period, all the XFG were weighed and slaughtered using exsanguination. The dressed weight, half-eviscerated weight and eviscerated weight were measured immediately after scalding while removing the breast muscle and leg muscle, which were weighed, and the data were recorded. Then, intestinal segments of 5 cm were collected from the middle part of the jejunum and ileum using aseptic manipulation and rinsed with 0.9% saline solution to clean the intestinal contents, and the samples were put into 4% paraformaldehyde to be fixed, facilitating the follow-up morphological analysis. Cecum contents were collected in sterile 5 mL polypropylene tubes in an environment with dry ice. Then, the samples were sent out for testing to perform DNA extraction.

### 2.4. Growth Performance Parameter Measurements

The average daily gain (ADG) of the geese in each group was calculated from the initial and final body weight (BW) (kg) of the geese in each group. The average daily feed intake (ADFI) (g) was calculated by recording the daily feed input for each group over the three weeks of the trial period. The remaining feed was calculated every seven days as the weekly feed intake, and then, the feed/gain (FCR) was calculated based on the average daily feed intake (ADFI) and average daily gain (ADG).

### 2.5. Slaughter Performance Parameter Measurements

Slaughter indicators including dressed yield (%), half-eviscerated yield (%), eviscerated yield (%), breast muscle yield (%) and leg muscle yield (%) were calculated according to the noun terms and metric statistics method (NY/T823-2020) for poultry production performance.

### 2.6. Histological Observation of Jejunum and Ileum

Intestine samples were embedded in paraffin using a standard procedure, sectioned into 7 µm slices and stained with hematoxylin and eosin (H&E) (Bio-Sail Biotech Technology Co., Ltd., Beijing, China). The villi height (VH) and crypt depth (CD) were measured for each sample under a light microscope (X20 panoramic scan) using the Slide Viewer (version 2.5.0) image analysis system. The ratio of villi height to crypt depth (VH/CD) was also calculated. Two XFGs with similar body weights were randomly selected from each replicate for intestinal section observation (a total of six samples). Five intact villi were randomly selected from each section for measurement, the VH and CD of a total of 30 intact villi in each group were recorded to calculate the VH/CD, and the total mean value was recorded. The measurement criteria and methods refer to those used by Wang et al. [16].

### 2.7. DNA Extraction and 16S RNA Gene Amplicon Sequencing

Total genomic DNA was extracted from the cecal contents using an OMEGA Soil DNA Kit (M5635-02) (Omega Bio-Tek, Norcross, GA, USA). Each step was performed according to the manufacturer’s instructions. Samples were then stored at −20 °C for further analysis. DNA concentration and purity were measured using a Nanodrop NC2000 spectrophotometer (Thermo Fisher Scientific, Waltham, MA, USA). In addition, the quality of the extracted DNA was determined using agarose gel electrophoresis. Then, the V3-V4 region of the 16S rRNA gene was amplified by PCR using forward primer 338F (5′-ACTCCTACGGGAGGCAGCA-3′) and reverse primer 806R (5′-GGACTACHVGGGTWTCTAAT-3′) [17].

Sample-specific 7-bp bar codes were incorporated into the primers for multiplex sequencing. The 250-bp paired-end amplicon libraries were sequenced using the Illumina NovaSeq platform (Illumina, San Diego, CA, USA). Microbiome bioinformatics was performed using QIIME2 2019.4 for sequence analysis [18]. Briefly, raw sequence data were demultiplexed using the demux plugin, followed by primer cutting with the cut adapt plugin [19]. The DADA2 plugin [20] was used for denoising and clustering. Sequence data analysis was performed using the QIIME2 and R packages (version 3.2.0). Alpha diversity indices (Chao1 richness estimates, Shannon’s diversity index, Simpson’s index and observed species) at the ASV (amplicon sequence variant) level were calculated using the ASV table and visualized as box plots. In addition, to study the structural variation in microbial communities between samples, weighted UniFrac distance metrics [21] were used to conduct beta diversity analysis and visualization through principal coordinate analysis (PCoA).

LEfSe software (version 1.0) was used to perform linear discriminant analysis (LDA) effect size analysis (LEfSe) to identify potential microbial biomarkers based on an LDA > 3 [22]. The prediction of microbial functional potential between groups by PICRUSt2 (Phylogenetic Investigation of Communities by Reconstruction of Unobserved States) was based on the databases of MetaCyc.

### 2.8. Data Analysis

We used SPSS (version 21.0; SPSS Inc., Chicago, IL, USA) to analyze all production performance data and intestinal morphology using ANOVA and Mann–Whitney tests, respectively. Group data are presented as mean ± standard error. Histogram graphs were produced using GraphPad Prism 9 software (San Diego, CA, USA). The correlations between phenotypes and differential flora and differential flora and differential metabolic pathway correlations were examined using QIIME2 and R (version 3.2.0) packages to evaluate Spearman’s rank correlation coefficient. *p* < 0.05 indicates significant differences (* *p* < 0.05; ** *p* < 0.01; *** *p* < 0.001).

## 3. Results

### 3.1. Growth Performance and Slaughter Performance

Table 2 reports the growth and slaughter performance of geese. Before the initiation of the experiment, there were no significant differences in BW among the groups. The BW of the geese in Group A (the control) and the 15% FMS-treated group were similar at the end of the experiment, and both were higher than that in the 5% and 10% FMS-treated groups. There were no significant differences among the four groups (*p* > 0.05). Compared with groups B and C, the ADG of Group D was significantly increased (*p* < 0.001), and the feed/gain ratio was significantly decreased (*p* < 0.001); however, there was no significant difference in ADFI among the four groups.

The percentage of dressed yield was significantly higher in Group D than in Group C (*p* < 0.05), and the percentage of eviscerated yield was significantly lower in Group B than in Groups A and D (*p* < 0.05; *p* < 0.05). There was no significant difference in the percentage of half-eviscerated yield, breast muscle yield, leg muscle rate and lean meat yield (edible portion) in the four groups (*p* > 0.05). Among the three experimental groups, Group D was superior to the other two groups in all slaughter performance indexes. Furthermore, the percentage of dressed yield, eviscerated yield and breast muscle rate were improved compared to Group A. However, the differences were not significant (*p* > 0.05).

### 3.2. Intestinal Morphology of Jejunum and Ileum

**Figure 1:**
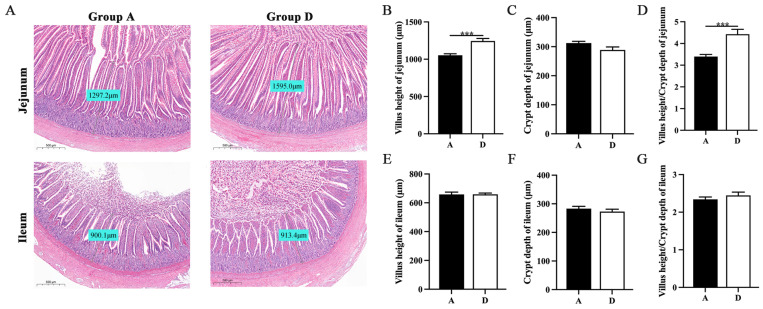
Morphology of jejunum and ileum in 15% FMS group and the control group. (**A**) Jejunum and ileum morphological observation of geese. (**B**–**D**) Jejunum morphological analysis of geese. (**E**–**G**) Ileum morphological analysis of geese. Each bar represents mean ± SEM. *** *p* < 0.001 indicates significant differences between Groups A and D.

Based on the above results, Group D, with the 15% addition of FMS, was selected for follow-up experiments. Jejunum and ileum mucosal morphology were observed using H&E staining under an optical microscope, as presented in Figure 1A. Histological analysis of the jejunum showed that Group D had a higher villi height (*p* < 0.001), lower crypt depth (*p* > 0.05) and higher VH/CD (*p* < 0.001) compared to Group A (Figure 1B–D). Histological analysis of the ileum showed that the ileal intestinal morphology of Group D was better than Group A under histological analysis. There is no significant difference in villi height, crypt depth and VH/CD in Group D compared to Group A (*p* > 0.05) (Figure 1E–G).

### 3.3. ASV Composition and Alpha and Beta Diversity Analysis of Caecum Flora

**Figure 2:**
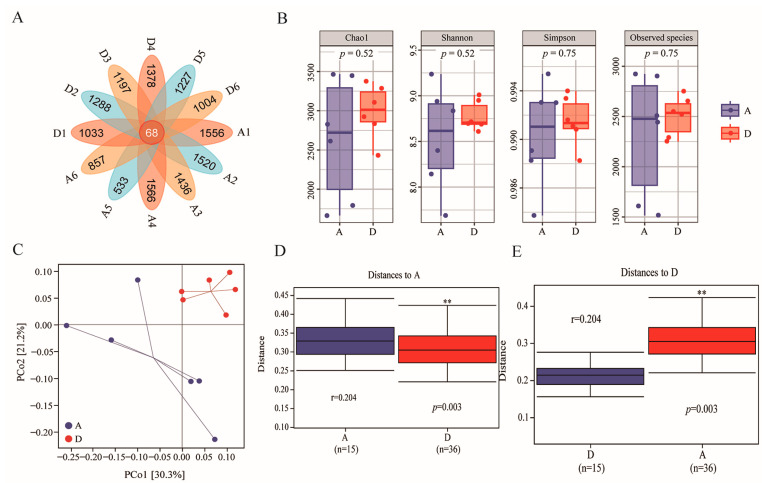
Plot of the results of microbiological sequencing analysis of the cecum. (**A**) Petal plot. (A1–A6 and D1–D6 represent six samples in groups A and D, respectively). (**B**) Alpha diversity index. (**C**) Two-dimensional plot of PCoA analysis based on weighted Unifrac distance. (**D**, **E**) Analysis of between-group differences obtained based on weighted Unifrac distances and PERMANOVA (adonis) test analysis. ** *p* < 0.01 indicates significant differences between Groups A and D.

To evaluate the effect of the fattening diet supplemented with 15% FMS on the intestinal microbiota of geese, 16S rRNA gene sequencing was performed on the cecal content; the sequencing depth detail of the caecum flora is shown in Supplementary Table S1. These reads were assigned using DADA2 in QIIME2. A total of 19,762 ASVs were obtained from 12 samples, and 68 ASVs were shared among the samples (Figure 2A).

Observed species, Chao1 and the Simpson and Shannon indices of alpha diversity were evaluated; there was no significant difference for all four alpha diversity indices (*p* > 0.05) between Groups D and A (Figure 2B). However, the median line of the box plot in Group D was higher than in Group A, and the entire distribution of Group D was shifted upward relative to Group A. Thus, we believe that the addition of 15% FMS helped to increase the α diversity of the colonies. Meanwhile, principal coordinates analysis (PCoA) based on the weighted Unifrac distance reveals that bacterial communities differed between Group D and Group A (r = 0.204, *p* = 0.003) (Figure 2C–E).

### 3.4. Taxonomic Composition and Clustering of Intestinal Flora

**Figure 3:**
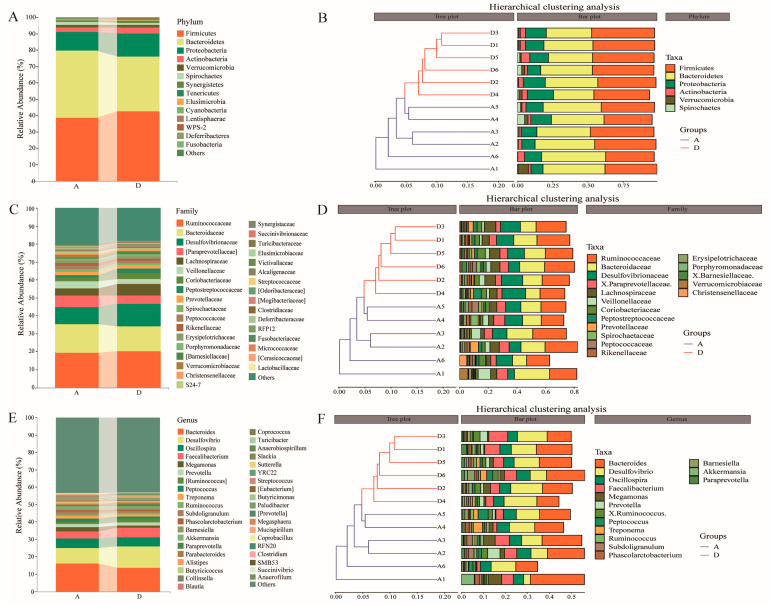
Dominant intestinal bacterial taxa and hierarchical clustering at the level of phylum, family and genus. (**A**) Community bar plot of bacteria with average relative abundance > 0.1% at the phylum level. (**B**) The hierarchical clustering analysis of bacteria with average relative abundance > 1% at the phylum level. (**C**) Community bar plot of bacteria with average relative abundance > 0.1% at the family level. (**D**) The hierarchical clustering analysis of bacteria with average relative abundance > 1% at the family level. (**E**) Community bar plot of bacteria with average relative abundance > 0.1% at the genus level. (**F**) The hierarchical clustering analysis of bacteria with average relative abundance > 1% at the genus level.

Taxonomic classification of intestinal flora was assigned to clarify the effects of 15% FMS on gut microbiota at the phylum, family and genus taxonomic levels, respectively. A total of 22 phyla, 113 families and 155 genera were detected in the cecal microorganisms of Groups A and D. Taxonomic analysis shows that the dominant bacteria of cecal microorganisms in Groups A and D were roughly similar, with *Firmicutes, Bacteroidetes* and *Proteobacteria* being the dominant phyla, accounting for >90% of the total. *Ruminococcaceae*, *Bacteroidaceae*, *Desulfovibrionaceae*, [*Paraprevotellaceae*], *Lachnospiraceae*, *Veillonellaceae* and *Coriobacteriaceae* were the dominant families, accounting for >60% of the total. *Bacteroides*, *Desulfovibrio*, *Oscillospira and Faecalibacterium* were the dominant genera. Clustering of the dominant phyla, family and genera (top 6, top 17 and top 15, respectively), with an average relative abundance > 1%, revealed that the clustering of Groups A and D formed a large branch, indicating that the overall compositional structure of the community had some similarity, while Groups A and D also formed independent clustering branches, respectively, indicating that the two groups differed in their overall compositional structure. Moreover, the Firmicutes/Bacteroidetes ratio of the cecum was slightly greater in Group D (1.27) than in Group A (0.93) (Figure 3A–F; Supplementary Table S2).

### 3.5. LDA Effect Size (LEfSe) and Correlation Analysis of Phenotypic Parameters and Differentially Enriched Microbes

**Figure 4:**
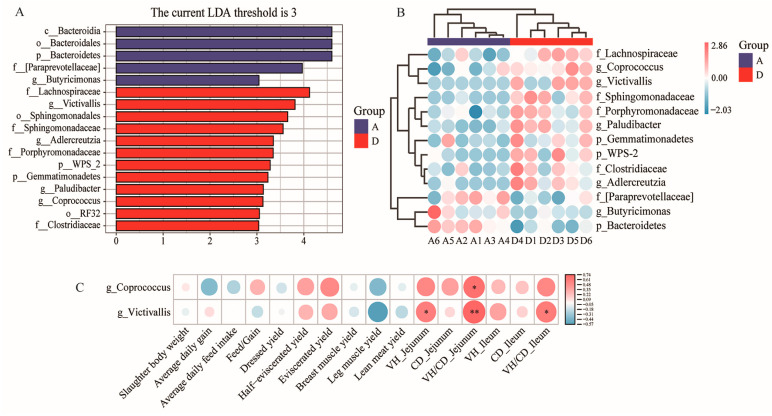
LEfSe analysis and correlation analysis. (**A**) Distribution of linear discriminant analysis (LDA) scores for cecal microorganisms between Groups A and D. (**B**) The interactive heat map only visualizes the distribution of differential bacterial abundance at the phylum, family and genus level between the samples from Groups A and D. (**C**) Correlation analysis between phenotypic parameters of the production performance, intestinal histomorphology and differentially enriched fecal microbes. * indicates *p* < 0.05; ** indicates *p* < 0.01. Abbreviations: VH, villus height; CD, crypt depth.

Differentially abundant taxa and specific biomarkers were identified using LEfSe (*p* < 0.05, LDA score > 3). The results show that the abundance of *Bacteroidia* (class), *Bacteroidales* (order), *Bacteroidetes* (phylum), [*Paraprevotellaceae*] (family) and *Butyricimonas* (genus) were lower in Group D than in Group A, whereas the abundance of *Lachnospiraceae* (family), *Victivallis* (genus), *Sphingomonadales* (order), *Sphingomonadaceae* (family), *Adlercreutzia* (genus), *Porphyromonadaceae* (family), *WPS-2* (phylum), *Gemmatimonadetes* (phylum), *Paludibacter* (genus), *Coprococcus* (genus), *RF-32* (order) and *Clostridiaceae* (family) were higher in Group D than in Group A (Figure 4A). The differential bacterial abundance at the phylum, family and genus level between Groups A and D was conveniently visualized using a heatmap (Figure 4B). Spearman’s correlation coefficient was used to evaluate the relationships between the production performance traits, intestinal histomorphology parameters and the relative abundance of differentially enriched bacteria in geese (Figure 4C). The results indicate that *Coprococcus* and *Victivallis*, which belong to the genus level, were positively correlated with VH/CD (jejunum) (*p* < 0.05; *p* < 0.01). Moreover, *Victivallis* was positively correlated with VH (jejunum) (*p* < 0.05) and VH/CD (ileum) (*p* < 0.05).

### 3.6. Functional Potential Prediction of the Intestinal Microbiota

**Figure 5:**
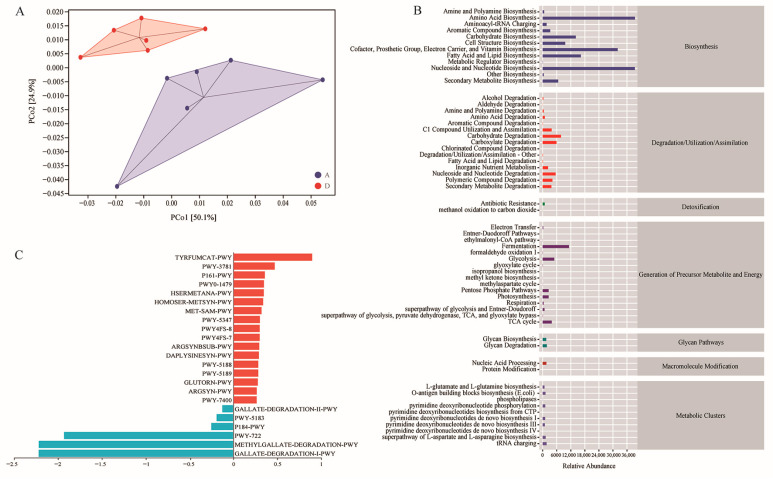
Functional potential prediction based on MetaCyc database. (**A**) PCoA analysis of functional units. (**B**) Statistics on metabolic pathways. (**C**) Difference analysis of metabolic pathways (*p* < 0.05). Red represents upregulated pathways, and blue represents downregulated pathways. CTP, cytidine triphosphate; *E. coli*, *Escherichia coli*; TCA, tricarboxylic acid cycle; tRNA, transfer ribonucleic acid.

To further assess the gut microbiotas’ functional properties, overall changes in function were analyzed using principal coordinate analysis (PCoA). The results show that the biological function of the intestinal flora of Group D was largely separated from that of Group A (Figure 5A). Next, we compared MetaCyc pathway abundance to explore the gut microbiome functionality between the two groups. A total of 60 pathways were associated with the experimental groups through the MetaCyc database. These MetaCyc pathways mainly covered fermentation, glycolysis, amino acid biosynthesis and carbohydrate biosynthesis/degradation (Figure 5B). Moreover, 23 key differential pathways were obtained (*p* < 0.05) (Figure 5C), including 17 upregulated pathways and 6 downregulated pathways. Among these, multiple amino acid biosynthesis, tRNA processing and aerobic respiration I (cytochrome c) were significantly upregulated. These findings reveal that the FMS diet alters the composition of the gut microbial community and function, thus affecting the metabolic activities of Xianghai flying geese.

### 3.7. Differential Bacteria of Gut Microbiota and Differential Metabolic Pathway Enrichment Analysis

**Figure 6:**
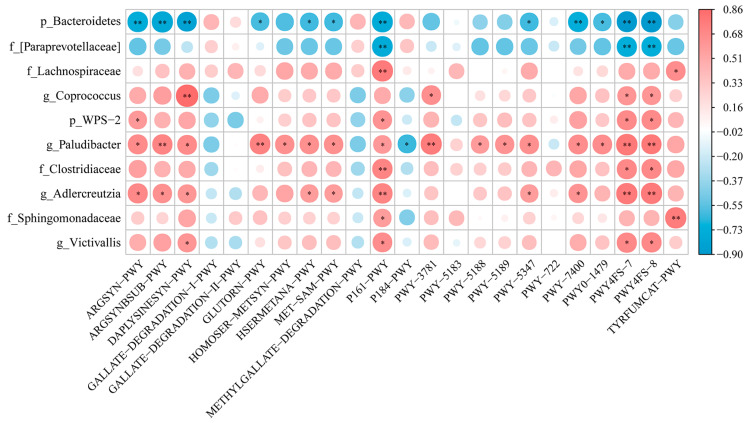
Heatmap of the association of differentially enriched metabolic pathways and differentially enriched fecal microbes. * indicates *p* < 0.05; ** indicates *p* < 0.01.

Spearman’s correlation analysis was further performed to interpret the relevance between differentially enriched bacteria and differential metabolic pathways (Figure 6). The results reveal that DAPLYSINESYN-PWY related to L-lysine biosynthesis I (*p* < 0.01; *p* < 0.05), PWY4FS-7 related to phosphatidylglycerol biosynthesis I (plastidic) (*p* < 0.05) and PWY4FS-8 related to phosphatidylglycerol biosynthesis II (non-plastidic) (*p* < 0.05) were positively correlated with the differential bacteria *Coprococcus* and *Victivallis* in genus, respectively. In contrast, *Bacteroidetes* (phylum) was negatively associated with 12 pathways, including ARGSYN-PWY, ARGSYNBSUB-PWY, DAPLYSINESYN-PW, etc., which are mainly associated with amino acid biosynthesis. Furthermore, the results show that most of the beneficial bacteria (*WPS-2*; *Paludibacter*; and *Adlercreutzia*) were significantly and positively correlated with the P161-PWY, PWY4FS-8, and PWY4FS-7 pathways. Overall, most of the altered metabolic pathways were associated with the composition of gut microbiota, indicating that FMS can activate some key physiological and metabolic activities that lead to the growth and development of geese.

## 4. Discussion

Fermentation can improve the palatability and nutritional content of diets, and enhancing the digestion of animals may result in improved weight gain [23]. FMS was chosen as a partial substitution for the basal fattening diet in this study. Similar to corn straw silage (CSS), FMS also has dietary fiber [24]. These dietary fibers can stimulate peristalsis in the gastrointestinal tract, which, in turn, regulates the digestion and absorption of nutrients [25]. Growth performance results show a significant decrease in FCR and a significant increase in ADG in the 15% FMS group compared to the other experimental groups. This result is in agreement with Yan et al., who found that the ADG of poultry increased after increasing the proportion of fermented feed added within a certain range. Specifically, the addition of 7.5% fermented feed resulted in an increase in ADG compared to 2.5% of the fermented feed group [26]. In addition, a pleasant surprise is that replacing a dietary portion with 15% FMS still performed well, similar to the control group. This indicates that the 15% FMS may have facilitated better nutrient digestion and absorption for geese while reducing the feed dosage, explaining the improved efficiency of feed utilization. There are also studies indicating that fermenting straw produces fulvic acid, which has some stimulating effects on growth, and the level of growth stimulation is related to an increase in the straw ratio [27]. This finding may also be an explanation for the increased FMS levels in the diet that can stabilize and maintain production performance. In addition, condensed molasses fermentation solubles (CMSs) are a resource with high crude protein content that can replace some concentrates in cow diets [27]. An appropriate proportion of CMS is beneficial to production enhancement, and both too low and too high levels negatively affect the production performance of cows. Therefore, evaluating the appropriate proportion of FMS in the XFG diet is important. The slaughter performance results also show that the 15% FMS group significantly improved the dressed yield and eviscerated yield of XFG compared to the 5% FMS group and 10% FMS group. There was no significant difference in various slaughter performance indicators between the 15% FMS group and the control group. Among them, the dressed yield, eviscerated yield and breast muscle yield in the 15% FMS group performed better than the control group. This result indicates that 15% FMS as a feed substitute may support higher animal performance and effectively improve the slaughter performance of XFG.

Gut health is one of the major factors governing poultry performance; therefore, improving gut health is essential for poultry welfare and productivity [28]. Of these, intestinal villus height (VH), crypt depth (CD) and VH/CD are important indices for measuring intestinal digestion and absorption capacity [29]. Higher VH and VH/CD represent a higher intestinal nutrient absorption capacity [30]. Dietary components affect the gut health of healthy poultry. This report shows that feeding fermented feed to broilers can increase VH/CD and improve the absorption area and villus height in the jejunum [31]. It is well known that the digestive tract of geese has strong motility and rotation motility [32], which can promote the decomposition of dietary cellulose. An amount of 15% FMS was used to replace feed for XFG with more tolerant roughage characteristics in this study. The jejunum and ileum have higher VH and VH/CD, indicating a positive change in the intestinal morphology of XFG. This increase may be the reason for the significant effects of dietary treatment with 15% FMS on the growth and slaughter performance of XFG. Therefore, the 15% FMS group of XFG has stronger intestinal absorption ability, making them more adaptable to diet, environment and diseases.

Cecal microbiota plays a key role in the production performance of geese. The β diversity analysis shows that 15% FMS had a certain impact on geese microbial composition. Some reports claim that feeding a high-fiber diet to geese leads to an increase in *Firmicutes* and a decrease in *Bacteroides* [33], which is consistent with the results of this study. In addition, *Firmicutes*/*Bacteroides* are crucial for maintaining a normal intestinal balance due to the complementary symbiotic relationship between them [34]. In this study, the *Firmicutes*/*Bacteroides* of the 15% FMS group in the cecum were higher than in the control group, increasing the potential for energy uptake or storage in XFG. Short-chain fatty acids (SCFAs) mainly contain acetate, propionate and butyrate. They are products of fiber fermentation that are produced by the intestinal flora. SCFAs improve host energy metabolism and the inflammatory response [35]. *Lachnospiraceae* are a potentially beneficial bacteria that were abundant in the experimental group in this study. Also, they has the ability to produce acetic acid and butyric acid in the intestine and are beneficial for the proliferation, differentiation and overall health of animal intestinal epithelial cells [36]. This finding explains why energy metabolism levels in the 15% FMS group did not decrease. *Coprococcus* belongs to *Lachnospiraceae,* which are known to break down carbohydrates into SCFAs [37]. *Victivallis* has fiber disaccharide degradation activity, which contributes to the production of acetate salts [38]. They were all upregulated in the 15% FMS group and significantly positively correlated with VH/CD in jejunum. This finding indicates that FMS promotes the generation of SCFAs and has a positive effect on improving intestinal morphology. In addition, some other beneficial bacteria were also found in the experimental group, such as *Paludibacter* (a propionate producer) [39].

In addition to affecting the composition of intestinal microbiota, FMS also alters the metabolism of cecal microbiota. Research has found that under a healthy dietary pattern, the abundance of phosphatidylglycerol biosynthesis (PWY4FS-7 and PWY4FS-8) and acetylene degradation (P161-PWY) pathways in the gut microbiota is higher [40], which is consistent with the results of this experiment. Moreover, the above pathways were significantly and positively correlated with the beneficial bacteria *Paludibacter* and *Victivalis*, which were significantly upregulated in the 15% FMS group. Amino acid metabolism is extremely important to support animal growth, maintain homeostasis and regulate other biological processes in the host and intestinal microbiota. Among them, arginine, methionine, ornithine and lysine are essential for improving animal growth performance and feed efficiency [41]. They are closely related to physiological processes such as immune function, protein synthesis and growth promotion [42] and help to enhance intestinal epithelial barrier functions [43]. Supplementation with methionine in diets improved the average daily gain and body weight of animals [44]. Ornithine is an important intermediate in the urea cycle, which is responsible for most nitrogen excretion by converting toxic ammonia into urea in the liver [45]. Lysine deficiency can decrease body weight because lysine is mainly used for muscle protein production in poultry [46]. The PICRUSt2 results show that the 15% FMS group significantly enhanced the L-arginine biosynthesis pathway (ARGSYNBSUB-PWY, ARGSYN-PWY and PWY-7400), L-methionine biosynthesis pathway (HSERMETANA PWY, HOMOSER METSYN-PWY, MET-SAM-PWY and PWY-5347) and L-ornithine biosynthesis pathway (GLUTORN-PWY). In agreement with previous literature reports, the present study demonstrates that dietary supplementation with 15% FMS significantly altered growth performance and upregulated the abundance of *Coprococcus* and *Victivalis* in the cecum of XFG by strengthening the L-lysine biosynthesis I pathway of bacteria. In addition to the changes observed in the above signaling pathway, we observed alterations in other metabolism pathways, including aerobic respiration (cytochrome c) (PWY-3781), tRNA processing (PWY0-1479) and tetrapyrrole biosynthesis (PWY-5188, PWY-5189). Therefore, FMS activates the metabolic pathways associated with growth and development, offering the potential to replace the feed of fattened geese. However, many metabolic regulatory pathways remain unknown, creating vast research potential.

Until now, there has been limited information about the regulation and function of poultry intestinal microbiota. More in-depth studies are needed to understand the effect of FMS on the gut microbiota of indigenous XFG. Studies have shown that an elevated ratio of *Firmicutes*/*Bacteroides* may be related to obesity [47,48]. However, abdominal fat was not measured in the present study due to limited experimental conditions; thus, we could not determine the effect of FMS on abdominal obesity. During the fattening period, 15% FMS may not be the optimal level for replacing the feed of XFG. However, in this experiment, the addition of 15% improved the abundance of cecal microbiota in XFG, reduced feed costs and exhibited no detrimental impacts on the growth and development of XFG. This research only provides FMS data based on experimental results in XFG. Hence, it only serves as a potential guideline for the partial replacement of fattening diets with FMS. Further research is needed to determine the optimal FMS level for the growth performance of fattened geese and the detailed mechanism of FMS. As gut microbiota function only offers a preliminary prediction, it is difficult to confirm whether these differential pathways actively participate in physiologically regulating the gut microbiota of XFG. In future research, we intend to isolate and culture microorganisms from fecal matter to investigate the distinct functional attributes of the gut microbiome. We anticipate that future research will support FMS as a viable alternative to partial fattening feed, thereby facilitating cost-effective feed utilization by establishing a causal relationship between FMS and gut microbiota composition and function.

## 5. Conclusions

This study was the first to analyze the effect of different fermented maize stover (FMS) percentages on the fattening phase of Xianghai flying geese. Adding 15% FMS to the basal fattening diet achieved similar levels of production performance and improved the intestinal morphology of geese compared to the control group. Furthermore, the cecum microbiota difference analysis shows that the composition of gut microbiota was significantly altered in the 15% FMS group and that the beneficial bacteria *Coprococcus* and *Victivallis* in the genus could promote VH/CD in the jejunum. Intestinal flora function analysis also reveals that DAPLYSINESYN-PWY related to L-lysine biosynthesis I, PWY4FS-8 related to phosphatidylglycerol biosynthesis II and PWY4FS-7 related to phosphatidylglycerol biosynthesis I are associated with growth performance in geese. The present results provide insights into applying subsequent FMS to the geese-fattening diets of Xianghai flying geese. Additionally, they provide a theoretical basis and practical reference value for using non-conventional feedstuffs in the aquaculture industry of poultry to perform feed substitution and reduce farming costs.

## Figures and Tables

**Table 1:** Formulations and nutritional levels of basal fattening diet and fermented maize stover.

Items	Basal Fattening Diet
Ingredient, %	
Corn grain	50.10
Sorghum grain	21.80
Soybean meal	13.00
Cottonseed meal	10.50
Limestone	0.90
Sodium bicarbonate	1.20
NaCl	0.40
DL-Methionine	0.10
^1^ Pre-mix	2.00
Total	100.00
Nutritional levels	
^2^ Metabolizable energy/(MJ/kg)	11.74
Crude protein (%)	15.25
Crude fiber (%)	3.69
Calcium (%)	0.85
Total phosphorus (%)	0.64
Available phosphorus (%)	0.36
Lysine (%)	0.72
Methionine (%)	0.36
	**Fermented maize stover**
Ingredient, %	
Dry matter	35.97
Organic substance	93.30
Crude protein	6.17
Crude fiber	40.65
Crude ash	6.70
Neutral detergent fiber	76.06
Acid detergent fiber	47.06
Lignin	7.74
^3^ Gross energy/(MJ/kg)	20.18
Digestible energy/(MJ/kg)	9.83
Metabolizable energy/(MJ/kg)	7.94
Fermentation quality parameters	
pH	3.96
Lactic acid (% DM)	4.41
Acetic acid (% DM)	1.23
Propionic acid (% DM)	0.021
Butyric acid (% DM)	^4^ ND
^5^ NH_3_-N/TN (%)	3.21

^1^ Supplied per kilogram of diet containing the following: vitamin A 30,000 IU, vitamin D3 5000 IU, vitamin E 40 mg, vitamin K3 4 mg, vitamin B1 3.2 mg, vitamin B2 13 mg, vitamin B6 6.5 mg, vitamin B12 0.3 mg, niacin 56 mg, pantothenic acid 16 mg, folic acid 1.6 mg, biotin 150 mg, Fe 100 mg, Cu 8 mg, Zn 100 mg, Mn 160 mg, I 2.6 mg, Se 36 mg. ^2^ Nutritional levels are calculated values. ^3^ Gross energy is based on calculated values; others are analyzed values. ^4^ ND means not detected. ^5^ NH_3_-N/TN, ammoniacal nitrogen/total nitrogen.

**Table 2:** Effects of FMS on the production performance in Xianghai flying geese.

Indicators	A	B	C	D	SEM	*p*-Value
Initial body weight (g)	3012.93 ± 35.844	3009.06 ± 35.427	2998.25 ± 34.802	2995.5 ± 35.369	17.597	0.982
Slaughter body weight (g)	3505.33 ± 37.734	3411.67 ± 36.866	3419.13 ± 31.393	3472.93 ± 33.101	17.492	0.180
Average daily gain (g)	23.45 ± 0.225 ^A^	19.17 ± 0.308 ^C^	20.04 ± 0.335 ^B^	22.73 ± 0.345 ^A^	0.184	<0.001
Average daily feed intake (g)	208.99 ± 0.396	208.26 ± 0.559	208.2 ± 0.172	208.57 ± 0.399	0.197	0.525
Feed/gain (g/g)	8.97 ± 0.078 ^C^	11.08 ± 0.188 ^A^	10.58 ± 0.159 ^B^	9.33 ± 0.141 ^C^	0.089	<0.001
Dressed yield (%)	87.39 ± 0.199 ^ab^	87.31 ± 0.139 ^ab^	87.09 ± 0.162 ^b^	87.64 ± 0.178 ^a^	0.086	0.159
Half-eviscerated yield (%)	78.66 ± 0.220	77.85 ± 0.429	78.25 ± 0.215	78.52 ± 0.297	0.152	0.247
Eviscerated yield (%)	71.19 ± 0.192 ^a^	70.19 ± 0.420 ^b^	70.69 ± 0.309 ^ab^	71.29 ± 0.287 ^a^	0.157	0.051
Breast muscle yield (%)	17.64 ± 0.161	17.16 ± 0.195	17.53 ± 0.154	17.66 ± 0.159	0.084	0.121
Leg muscle yield (%)	12.61 ± 0.132	12.4 ± 0.138	12.39 ± 0.109	12.55 ± 0.111	0.062	0.511
Lean meat yield (%)	30.24 ± 0.273	29.55 ± 0.321	29.92 ± 0.254	30.21 ± 0.252	0.138	0.257

^ABC^ Values and ^ab^ values with different superscripts within the same row indicate a significant difference at *p* < 0.001 and *p* < 0.05, respectively.

## Data Availability

The high throughput sequencing (HTS) datasets presented in this study are openly available in the NCBI’s Sequence Read Archive (SRA) with accession number PRJNA1047959. The data presented in this study are available in Supplementary Materials.

## Supplementary Materials

The following supporting information can be downloaded at: https://www.mdpi.com/article/10.3390/ani14030433/s1, Table S1: Sequence quality controls and DADA2 denoise of 16S rRNA sequencing data; Table S2: Comparison of the relative abundance of bacterial phylum, family and genus under group A and group D.
