# Supplementary material for: Optimization of Fermented Maize Stover for the Fattening Phase of Geese: Effect on Production Performance and Gut Microflora
Source: Animals (Basel). 2024 Jan 29;14(3):433. doi: 10.3390/ani14030433 (PMC10854615; doi:10.3390/ani14030433)
Supplement: Supplementary file 1 [file animals-14-00433-s001.zip › Supplementary Table S1.docx]

**Supplementary Table S1.** Sequence quality controls and DADA2 denoise of 16S rRNA sequencing data.

|  | **SampleID** | **Input** | **Filtered** | **Denoised** | **Merged** | **Non-chimeric** | **Non-singleton** |
| --- | --- | --- | --- | --- | --- | --- | --- |
| **A** | A1 | 128353 | 115756 | 112391 | 102908 | 66375 | 64572 |
|  | A2 | 128079 | 116862 | 112833 | 98137 | 68338 | 66106 |
|  | A3 | 102122 | 93180 | 89602 | 77729 | 53930 | 52267 |
|  | A4 | 136586 | 121566 | 117363 | 103386 | 73163 | 71168 |
|  | A5 | 107157 | 96327 | 94157 | 87996 | 72471 | 71960 |
|  | A6 | 86064 | 78704 | 76244 | 68283 | 48444 | 47477 |
| **D** | D1 | 93539 | 84142 | 80803 | 70411 | 53136 | 51880 |
|  | D2 | 121264 | 108905 | 105176 | 92150 | 67036 | 65200 |
|  | D3 | 134293 | 122409 | 118155 | 104027 | 77697 | 75901 |
|  | D4 | 143288 | 129473 | 124580 | 108353 | 82433 | 80600 |
|  | D5 | 134590 | 122506 | 118124 | 103673 | 77989 | 76090 |
|  | D6 | 141118 | 126982 | 123144 | 111000 | 85215 | 83913 |

After removing the primer fragment of the sequence and discarding the sequence of the mismatched primer by using QIIME2, Dada2 algorithm was called to process the feature sequence, for instance, quality control, de-noising, merging, de-chimerism, etc. The high-quality reads were summarized to generate a feature table with information about the abundance of ASVs in the samples and removed singletons ASVs. Using the R package, the lengths of all samples containing high-quality reads were counted.
