# Supplementary material for: Optimization of Fermented Maize Stover for the Fattening Phase of Geese: Effect on Production Performance and Gut Microflora
Source: Animals (Basel). 2024 Jan 29;14(3):433. doi: 10.3390/ani14030433 (PMC10854615; doi:10.3390/ani14030433)
Supplement: Supplementary file 1 [file animals-14-00433-s001.zip › Supplementary Table S2.docx]

Supplementary Table S2. Comparison of the relative abundance of bacterial phylum, family and genus under group A and group D.

| **Level** | **Bacteria** | **Group** | |
| --- | --- | --- | --- |
|  |  | **A** | **D** |
| Phyla | Firmicutes | 38.56% | 42.63% |
|  | Bacteroidetes | 41.29% | 33.44% |
|  | Proteobacteria | 11.27% | 14.03% |
|  | Actinobacteria | 2.62% | 3.77% |
|  | Verrucomicrobia | 1.81% | 1.37% |
|  | Spirochaetes | 1.73% | 1.42% |
|  | Synergistetes | 0.67% | 0.77% |
|  | Tenericutes | 0.34% | 0.34% |
|  | Elusimicrobia | 0.40% | 0.27% |
|  | Cyanobacteria | 0.12% | 0.47% |
|  | Lentisphaerae | 0.26% | 0.33% |
|  | WPS-2 | 0.05% | 0.39% |
|  | Deferribacteres | 0.13% | 0.19% |
|  | Fusobacteria | 0.26% | 0.02% |
|  | others | 0.50% | 0.54% |
| Familes | Ruminococcaceae | 19.06% | 20.03% |
|  | Bacteroidaceae | 16.09% | 13.80% |
|  | Desulfovibrionaceae | 9.56% | 12.79% |
|  | [Paraprevotellaceae] | 6.29% | 4.56% |
|  | Lachnospiraceae | 4.04% | 6.53% |
|  | Veillonellaceae | 4.01% | 2.65% |
|  | Coriobacteriaceae | 2.39% | 3.47% |
|  | Peptostreptococcaceae | 1.15% | 2.33% |
|  | Prevotellaceae | 1.65% | 1.80% |
|  | Spirochaetaceae | 1.67% | 1.35% |
|  | Peptococcaceae | 1.76% | 1.23% |
|  | Rikenellaceae | 1.54% | 1.27% |
|  | Erysipelotrichaceae | 1.68% | 1.09% |
|  | Porphyromonadaceae | 0.82% | 1.25% |
|  | [Barnesiellaceae] | 0.74% | 1.28% |
|  | Verrucomicrobiaceae | 1.30% | 0.64% |
|  | Christensenellaceae | 1.05% | 0.74% |
|  | S24-7 | 0.87% | 0.88% |
|  | Synergistaceae | 0.67% | 0.77% |
|  | Succinivibrionaceae | 0.76% | 0.22% |
|  | Turicibacteraceae | 0.21% | 0.49% |
|  | Elusimicrobiaceae | 0.40% | 0.27% |
|  | Victivallaceae | 0.26% | 0.33% |
|  | Alcaligenaceae | 0.28% | 0.28% |
|  | Streptococcaceae | 0.28% | 0.25% |
|  | [Odoribacteraceae] | 0.36% | 0.15% |
|  | [Mogibacteriaceae] | 0.30% | 0.17% |
|  | Clostridiaceae | 0.11% | 0.25% |
|  | Deferribacteraceae | 0.13% | 0.19% |
|  | RFP12 | 0.02% | 0.27% |
|  | Fusobacteriaceae | 0.25% | 0.01% |
|  | Micrococcaceae | 0.09% | 0.14% |
|  | [Cerasicoccaceae] | 0.00% | 0.11% |
|  | Lactobacillaceae | 0.04% | 0.05% |
|  | Others | 20.13% | 18.35% |
| Genus | Bacteroides | 16.08% | 13.80% |
|  | Desulfovibrio | 8.85% | 12.20% |
|  | Oscillospira | 5.56% | 5.06% |
|  | Faecalibacterium | 3.98% | 5.57% |
|  | Megamonas | 2.69% | 1.45% |
|  | Prevotella | 1.64% | 1.78% |
|  | [Ruminococcus] | 1.42% | 1.98% |
|  | Peptococcus | 1.76% | 1.23% |
|  | Treponema | 1.38% | 1.17% |
|  | Ruminococcus | 1.06% | 1.22% |
|  | Subdoligranulum | 1.43% | 0.76% |
|  | Phascolarctobacterium | 1.08% | 0.92% |
|  | Barnesiella | 0.73% | 1.27% |
|  | Akkermansia | 1.30% | 0.64% |
|  | Paraprevotella | 0.64% | 1.20% |
|  | Parabacteroides | 0.70% | 0.87% |
|  | Alistipes | 0.90% | 0.55% |
|  | Butyricicoccus | 0.53% | 0.56% |
|  | Collinsella | 0.36% | 0.45% |
|  | Blautia | 0.31% | 0.51% |
|  | Coprococcus | 0.26% | 0.47% |
|  | Turicibacter | 0.21% | 0.49% |
|  | Anaerobiospirillum | 0.61% | 0.06% |
|  | Slackia | 0.27% | 0.33% |
|  | Sutterella | 0.28% | 0.28% |
|  | YRC22 | 0.44% | 0.11% |
|  | Streptococcus | 0.28% | 0.24% |
|  | [Eubacterium] | 0.33% | 0.11% |
|  | Butyricimonas | 0.31% | 0.12% |
|  | Paludibacter | 0.07% | 0.30% |
|  | [Prevotella] | 0.09% | 0.28% |
|  | Megasphaera | 0.13% | 0.21% |
|  | Mucispirillum | 0.13% | 0.19% |
|  | Coprobacillus | 0.25% | 0.07% |
|  | RFN20 | 0.12% | 0.15% |
|  | Clostridium | 0.21% | 0.05% |
|  | SMB53 | 0.06% | 0.15% |
|  | Succinivibrio | 0.10% | 0.09% |
|  | Anaerofilum | 0.10% | 0.09% |
|  | Others | 43.37% | 43.02% |
